# Depression Symptoms in Haemodialysis Patients Predict All-Cause Mortality but Not Kidney Transplantation: A Cause-Specific Outcome Analysis

**DOI:** 10.1007/s12160-017-9918-9

**Published:** 2017-12-12

**Authors:** Joseph Chilcot, Ayman Guirguis, Karin Friedli, Michael Almond, Clara Day, Maria Da Silva-Gane, Andrew Davenport, Naomi A Fineberg, Benjamin Spencer, David Wellsted, Ken Farrington

**Affiliations:** 1 Health Psychology Section, Psychology Department, Institute of Psychiatry, Psychology and Neuroscience, King’s College London, London, UK; 2 Renal Unit, Lister Hospital, East & North Hertfordshire NHS Trust, Stevenage, UK; 3 Hertfordshire Partnership University NHS Foundation Trust, Rosanne House, Welwyn Garden City, UK; 4 Postgraduate Medical School, University of Hertfordshire, College Lane Campus, Hatfield, UK; 5 Centre for Lifespan and Chronic Illness Research, Department of Psychology, School of Life and Medical Sciences, University of Hertfordshire, College Lane Campus, Hatfield, UK; 6 Southend University Hospital NHS Foundation Trust, Prittlewell Chase, Westcliff-On-Sea, Essex, UK; 7 Department of Renal Medicine, University Hospitals Birmingham NHS Foundation Trust, Queen Elizabeth Hospital, Birmingham, UK; 8 UCL Centre for Nephrology, Royal Free Hospital NHS Foundation Trust, London, UK; 9 Department of Psychological Medicine, Institute of Psychiatry, Psychology and Neuroscience, King’s College London, Weston Education Centre, London, UK; 10 South London and Maudsley NHS Foundation Trust, Maudsley Hospital, Denmark Hill, London SE5 8AZ, UK

**Keywords:** Depression, Haemodialysis, Dialysis, Survival, Mortality, Outcome, Transplantation, Cause-specific hazard models

## Abstract

**Background:**

Depression is common in haemodialysis (HD) patients and associated with poor outcomes.

**Purpose:**

To evaluate whether depression symptoms predict survival and transplantation in a large sample of haemodialysis patients using cause-specific survival models.

**Methods:**

Survival data was collected between April 2013 and November 2015, as part of the screening phase of a multicentre randomised placebo-controlled trial of sertraline in HD patients. Depression was measured using the Beck Depression Inventory-II (BDI-II) and the Patient Health Questionnaire-9 (PHQ-9). Demographic and clinical data were collected via a self-report questionnaire and medical records. Competing risk survival analysis involved cause-specific and subdistribution hazard survival models. All models were adjusted for appropriate covariates including co-morbidity and C-reactive protein (CRP) in a subanalysis.

**Results:**

Of 707 cases available for analysis, there were 148 deaths. The mean survival time was 787.5 days. Cumulative survival at 12 months was 88.5%. During the study follow-up period, there were 92 transplants. The cumulative transplant event rate at 12 months was 7.8%. In separate adjusted models, depression symptoms predicted mortality (BDI-II HR = 1.03 95% CI 1.01, 1.04; PHQ-9 HR = 1.04 95% CI 1.01, 1.06). With respect to screening cut-off scores, a PHQ-9 ≥ 10 was associated with mortality (HR = 1.51 95% CI 1.01, 2.19) but not a BDI-II ≥ 16. Depression symptoms were not associated with time to transplantation in either cause-specific or subdistribution model.

**Conclusions:**

Consistent with past findings in HD patients, depression symptoms predicted survival but were not associated with kidney transplantation. Suitable treatments for depression need further evaluation, and their impact upon quality of life and clinical outcomes determined.

**Trial Registration Number:**

(ISRCTN06146268).

## Teaser

In kidney failure patients receiving haemodialysis, depression symptoms predict mortality but are not predictive of kidney transplantation.

## Introduction

Depression is a common comorbidity experienced across the spectrum of advanced kidney disease, particularly in End-Stage Kidney Failure (ESKF). Estimates of depression, as determined by cut-off scores from validated screening tools, suggest that approximately 39% of dialysis patients are depressed 1. This compares with around 23% when using diagnostic interviews 1. Not only is depression prevalent but it also contributes to a variety of poor outcomes, including increased hospitalisation and mortality amongst dialysis patients, a finding that has been well-documented within the literature [2–7]. For example, a recent meta-analysis reports that the presence of depressive symptoms in dialysis patients is associated with a 50% increase in the risk of mortality (HR = 1.51, 95% confidence interval 1.35–1.69) 4. The authors of this review conclude that there is considerable heterogeneity regarding the measurement of depression between studies, although overall, depression symptoms appear to be independent predictors of mortality. What is not clear from this review, however, was the number of studies which considered the presence of *competing risks*, a methodological issue which is addressed in the present study.

To elaborate, the examination of outcomes in ESKF is complicated by the presence of competing risks 8, which can include mortality, transplantation events and recovering renal function. A competing risk refers to “an event that either hinders the observation of the event of interest or modifies the chance that this event occurs” 8. Thus, in the context of examining survival in dialysis patients, transplantation is a competing event, since after transplantation the trajectory of health outcomes and risk of death is significantly altered. We are not aware of any previous studies examining the association between depression symptoms and survival in dialysis patients that have suitably handled the issue of competing risks.

In addition to considering the competing risks faced when evaluating outcomes in dialysis patients, the role of depression as a predictor of transplantation has not been extensively studied 9. Past work has shown that depression is associated with reduced odds of being on the kidney transplant waiting list but not a predictor of transplantation in those already on the waiting list 9. Since depression symptoms are amongst the most common of extra renal comorbidities, it is important to observe whether they are associated with transplantation, after adjustment for potential confounding factors such as age and physical comorbidities. Furthermore, since increased survival is associated with transplantation compared with dialysis 10, depression might be associated with survival in ESKF due to the reduced likelihood of receiving a kidney transplant. Currently, there is not enough data to examine the relationship between these outcomes.

The aims of this study were two-fold: (1) observe the causal-specific association between depression severity with mortality and transplant outcomes using two depression screening tools and (2) observe these relationships using prevalidated screening cut-off scores, indicating significant depressive symptomatology.

## Methods

### Design

This prospective survival analysis utilised depression screening data (*n* = 709) to select patients into a multicentre placebo-controlled feasibility randomised control trial (RCT) of sertraline in HD patients with mild to moderate Major Depressive Disorder 11. The full RCT protocol (trial registration number: ISRCTN06146268) 11 and the outcome paper 12 have been published elsewhere.

Patients were first screened for depression using two validated symptom severity measures (*n* = 709). Following screening of 709 HD patients, 231 patients (32.3%) had a Beck Depression Inventory-II (BDI-II) score of 16 or above. Sixty-three patients were eligible for further study and consented to be seen by the study psychiatrist, of which 37 (58.7%) were diagnosed with major depressive disorder (MDD). Thirty patients consented to be randomised, with 15 patients being randomised to sertraline and 15 to placebo groups. Patients were follow-up for 6 months as part of the original RCT.

The study reported here observed mortality and transplantation outcomes (i.e. receipt of a kidney transplant) from the point of depression screening (earliest entry 1st April 2013) until 1st November 2015 (end of follow-up). The screening period lasted until the end of October 2014. The date of completion of baseline screening data served as time zero in the survival models reported here. Outcome data was collected by medical records review, conducted by local study nephrologists. The median follow-up time was 579 days (interquartile range = 364).

### Patients

Patients were selected from the prevalent adult HD patients treated in five UK dialysis centres (see Friedli et al. 11 for full details of the original RCT). Adult ESKF patients who had been on HD for at least 3 months and who could speak and read English well enough to complete the questionnaires were eligible to be screened for depression. Patients provided consent for the research team to access their medical notes over the study duration. We also sought and had approved an ethics amendment to allow us to collect this outcome data.

### Depression Measures

Depression symptoms were measured using two validated screening tools: (1) Beck Depression Inventory-II (BDI-II) 13 and (2) Patient Health Questionnaire-9 (PHQ-9) 14. Both measures have shown validity for use within dialysis patients [15–17]. As well as continuous total scores, cut-off scores were also used to indicate significant depressive symptoms (PHQ-9 score ≥ 10 and a BDI-II ≥ 16) [15, 16]. In the present study, both the BDI-II and PHQ-9 total scores had high internal reliabilities (α = 0.93 and α = 0.88, respectively).

### Clinical and Demographic Factors

Demographic information was collected through a self-report questionnaire. Routinely collected clinical data was recorded from medical records, which included the comorbidities (presence of diabetes, heart disease, stroke, cancer, limb amputation, liver disease, lung disease), dialysis vintage (length of time on dialysis), haemoglobin (g/L), serum albumin (g/L), dialysis treatment adequacy (Kt/V) and dry weight (kg). C-reactive protein (CRP, mg/L), a marker of inflammation, was available in a subset of the sample (*n* = 396) since it is not routinely measured.

## Statistical Methods

All analysis was conducted in STATA 11.2. Separate cause-specific hazard survival models, using Cox regression, were evaluated for both survival and transplantation outcomes. Patients were censored for the alternative (competing) event. In addition, censorship occurred in relation to lost to follow-up, switching dialysis modality and recovering renal function. Using this approach, Hazard Ratios (HRs) are interpreted as “among those patients who did not (yet) experience the event of interest or competing event” (see Noordzij et al. 8). For example, if age predicted survival with a HR of 1.05, it would be interpreted as “in those who did not experience the event of interest (death) or a competing event (transplantation), a year increase in age is associated with a 5% increase in the hazard of death”.

The analysis was repeated using subdistribution hazard models using the approach proposed by Fine and Gray 18. This analysis provides estimates of the *probability* of an event (e.g. mortality) after considering a competing event (e.g. transplantation). Subdistribution Hazard Ratios (SHRs) that result from this method cannot be interpreted as an HR. Rather, SHRs represent the ratio in a “non-existing population including those who experience the competing event” 8. Put another way, a SHR for survival reflects the “mortality rate ratio among patients who are alive or have been transplanted before” 8.

In adjusted analysis, covariates were selected from clinical and demographic variables that showed univariate associations with the two outcomes of interest. For survival, the following covariates were adjusted for age, ethnicity (white vs. non-white), heart disease (present vs. not present), amputation of limbs (yes vs. no), diabetes (yes vs. no), haemoglobin (g/L), serum albumin(g/L), dialysis adequacy, dry weight (kg) and dialysis centre (dummy coded). With regard to transplantation, the following covariates were adjusted for age, ethnicity, heart disease, diabetes, haemoglobin, serum albumin, number of past kidney transplants and dialysis centre.

In all sets of analysis, the impact of depression symptoms upon the outcomes was evaluated in separate adjusted models that considered depression severity as a continuous score (either the PHQ-9 or BDI-II total score) or a measure-specific cut-off score (either a PHQ-9 score ≥ 10 or BDI-II ≥ 16). These cut-off scores were entered as a binary independent variable (i.e. depressed yes vs. no).

CRP was available in a subsample, since it is not routinely measured. Since CRP is associated with survival in dialysis patients [19, 20], we rerun the models in this subsample controlling for the respective covariates above and CRP (>5 mg/ L). In addition, we also explored the potential interaction between depression and ethnicity (white vs. non-white) upon survival since past evidence has suggested a moderated effect in kidney disease patients 21.

We report statistical significance using the standard *p* < 0.05 criteria. In addition, given that for each analytical method and outcome we treated depression symptoms using four methods, an adjusted *p* value cut-off (0.013) is also reported.

## Results

### Patient Characteristics

A summary of demographic and clinical characteristics is shown in Table 1. The majority of the sample was male (63.3%), and the average age was 64.1 (16.4) years. The median dialysis vintage was 33 months (interquartile range = 59). Mean depression scores at baseline were 13.5 (s.d. = 11.4) and 6.9 (s.d. = 6.2) for the BDI-II and PHQ-9, respectively. A total of 33.2% (95% confidence interval 30–37) had a BDI-II ≥ 16, and 28.1% (95% confidence interval 25–31) scored ≥10 on the PHQ-9. As expected, there was a strong association between the proportion of patient meeting both these thresholds (Chi-square = 375, *p* < 0.001). Furthermore, both the PHQ-9 and BDI-II total scores correlated highly (*r* = 0.86, *p* < 0.01).

**Table 1:** Summary of patient characteristics

Variable	Statistic
Age (mean, s.d.)	64.1 (16.4)
Gender (male, %)	63.3
Ethnicity (white, %)	69.0
Dialysis vintage (median, IQR) Number of past transplants	33 (59)
None	84%
1	14%
2	2%
Heart disease (%)	31.7
Diabetes (%)	33.3
Cancer (%)	10.6
Liver disease (%)	2.4
Lung disease (%)	6.4
Amputation of limbs (%)	3.2
Stroke (%)	8.0
Haemoglobin (g/L) (mean, s.d.)	11.1 (1.2)
Serum albumin (g/L) (mean, s.d.)	37.4 (4.4)
Dry weight (kg) (mean, s.d.)	75.5 (18.3)
CRP^a^ (>5 mg/L, %)	52%
Kt/V (mean, s.d.)	1.4 (0.3)

a Subsample (*n* = 396)

*IQR* interquartile range

In the subsample where CRP was available (*n* = 396), 52% had a CRP > 5 mg/L. Comparing those patients with CRP data and those without revealed no significant differences with regard to depression scores or the proportion scoring positively (BDI-II ≥ 16 and PHQ-9 ≥ 10).

### Survival Data and Outcomes

Of 707 cases available for analysis, there were 148 deaths. The estimated mean survival time was 787.5 days (s.e. = 10.4). Cumulative survival at 12 months was 88.5%. During the study follow-up period, there were 92 transplants (estimated mean survival time was 837.0 days, s.e. = 9.0). The cumulative transplant event rate at 12 months was 7.8% (by 12 months, 7.8% of the sample had received a transplant). The following censorship events were observed: lost to follow-up (*n* = 6), recovered renal function (*n* = 2) and switched dialysis modality (*n* = 1).

### Covariate-Adjusted Causal-Specific Hazard Models: Association of Depression Symptoms upon Mortality

**Figure 1:**
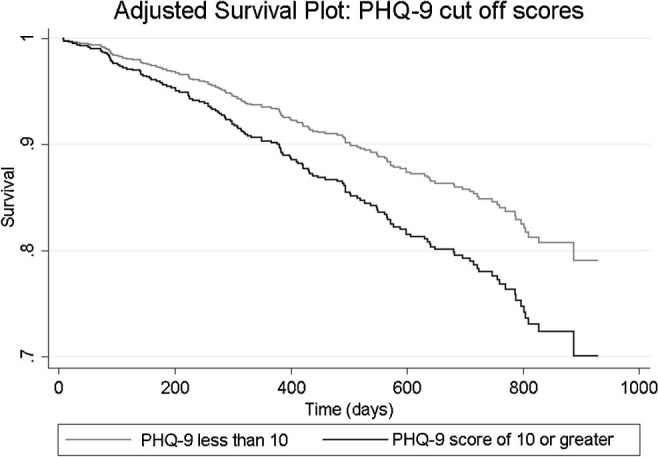
Adjusted survival plot showing hazard functions for patients with PHQ < 10 and PHQ-9 ≥ 10

In separate adjusted models, both the PHQ-9 and BDI-II predicted mortality (Table 2). In those who did not experience the event of interest (death) or a competing event (transplantation), a 1-point increase on the BDI-II and PHQ-9 was associated with a 3 and 4% increase in the hazard of death, respectively. With respect to screening cut-offs, only the PHQ-9 (≥10) was significantly associated with mortality (HR = 1.51, Fig. 1). A BDI ≥ 16 was not significantly associated with mortality (HR = 1.43, *p* = 0.055), although the effect size was similar to that of a PHQ-9 ≥ 10. After applying adjusted *p* value criteria, only the effect of the BDI-II and PHQ-9 continuous scores remained significant (Table 2).

**Table 2:** The association between depression with mortality and transplantation outcomes: adjusted cause-specific and subdistribution models

	Cause-specific models (HRs and 95% CI)		Subdistribution competing risks model(SHRs and 95% CI)	
	Mortality^a^	Transplantation^b^	Mortality^a^	Transplantation^b^
Full sample (*n*=707)				
BDI	1.03 (1.01, 1.04)^**^^[*]^	0.98 (0.96, 1.01)	1.03 (1.01, 1.04)^**^^[*]^	0.98 (0.96, 1.01)
PHQ-9	1.04 (1.01, 1.06)^**^^[*]^	0.98 (0.94, 1.02)	1.04 (1.01, 1.06)^**^^[*]^	0.97 (0.93, 1.01)
BDI ≥ 16	1.43 (0.99, 2.06)	0.84 (0.54, 1.32)	1.47 (1.02, 2.11)^*^	0.82 (0.52, 1.29)
PHQ-9 ≥ 10	1.51 (1.04, 2.19)^*^	0.85 (0.52, 1.38)	1.54 (1.06, 2.24)^*^	0.83 (0.50, 1.35)
CRP adjusted sub-analysis (*n* = 396)				
BDI	1.03 (1.01, 1.05)^*^	0.99 (0.96, 1.01)	1.03 (1.01, 1.06)^*^	0.99 (0.96, 1.01)
PHQ-9	1.04 (1.00, 1.10)^*^	0.97 (0.93, 1.01)	1.05 (1.01, 1.10)^*^	0.97 (0.91, 1.62)
BDI ≥ 16	1.54 (0.87, 2.73)	0.77 (0.44, 1.35)	1.61 (0.91, 2.90)	0.76 (0.43, 1.32)
PHQ-9 ≥ 10	1.70 (0.94, 2.90)	0.78 (0.43, 1.40)	1.73 (0.97, 3.11)	0.76 (0.41, 1.40)

a Model adjusted for age, ethnicity (white vs. non-white), heart disease (present vs. not present), amputation of limbs (yes vs. no), diabetes (yes vs. no), haemoglobin (g/L), serum albumin (g/L), dialysis adequacy, dry weight (kg) and dialysis centre (dummy coded)

b Model adjusted for age, ethnicity, heart disease, diabetes, haemoglobin, serum albumin, number of past kidney transplants and dialysis centre

CRP-adjusted models controlled for the relevant covariates listed above in addition to CRP > 5 (binary variable)

HR hazard ratio, SHR subdistribution hazard ratio, CI confidence interval

* *p* < 0.05

** *p* < 0.01

[*] indicates significance following adjusted *p* value cut-off (*p* < 0.013)

In subanalysis where CRP data was available, both BDI-II and PHQ-9 scores were associated with survival in adjusted models (Table 2). These effects were non-significant after applying adjusted *p* value criteria. Neither depression cut-off scores were associated with survival. All models were rerun examining the interaction (moderation) between depression and ethnicity upon survival. There was no significant interaction between depression (measured both as a continuous and cut-off score for the respective tools) and ethnicity upon mortality in either unadjusted or adjusted model (including CRP-adjusted subanalysis).

### Covariate-Adjusted Causal-Specific Hazard Models: Association of Depression Symptoms upon Transplantation

**Figure 2:**
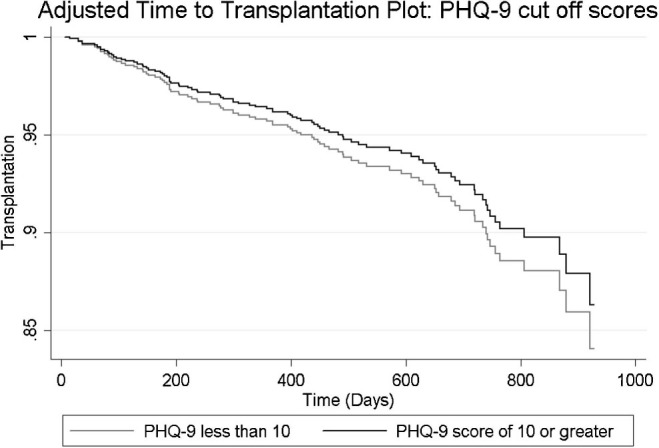
Adjusted survival (time to transplantation) plot showing hazard functions for patients with PHQ < 10 and PHQ-9 ≥ 10

Depression symptoms, treated as either a continuous total score or cut-off score, were not associated with transplantation events (see Fig. 2 for PHQ-9 cut-off score hazard functions). In the subanalysis that also controlled for CRP, the same null effects were observed (Table 2).

### Completing Risk Subdistribution Hazard Models

In addition to the causal-specific models, risk subdistribution hazard models were also evaluated for both mortality and transplantation (Table 2). For mortality, depression symptoms, treated as either a continuous total score or cut-off score, were associated with a greater cumulative incidence of death. In these models, the SHRs reflect the mortality ratio amongst patients who are alive or have been transplanted. However, after applying adjusted *p* value criteria, only the SHRs for the BDI-II and PHQ-9 continuous scores remained significant (Table 2). Similar to the cause-specific models, there were no associations between depression symptoms and kidney transplantation.

## Discussion

The aims of this paper were to examine the causal-specific associations between depression symptoms with both survival and kidney transplantation outcomes. We are not aware of any similar studies examining depression and mortality in dialysis patients where the competing event of transplantation has been considered.

Firstly, we found that approximately one in three patients had significant depressive symptoms as indicated by screening cut-offs of the BDI-II and PHQ-9. These results are in line with previous research 1 and further highlight that depressive symptoms are commonly experienced in dialysis patients.

Our findings here corroborate with past research showing that depression predicts all-cause mortality amongst dialysis patients 4. However, unlike our past work in incident dialysis patients 2, we failed to show that a BDI cut-off score ≥ 16 was significantly associated with survival, although a PHQ-9 score ≥ 10 was associated with an increased risk of death with a comparable effect size observed in a recent meta-analysis 4. After adjusted *p* value criteria were applied however, only the effect of the BDI-II and PHQ-9 continuous scores remained significant. It is possible that BDI-II and PHQ-9 cut-offs were not significant due to loss of information that occurs when dichotomizing the continuous predictor.

A strength of our study is the consideration of the competing influence of transplantation by using adjusted cause-specific survival models. We found that depressive symptoms, as measured by two validated screening tools (BDI-II and PHQ-9), were not predictive of transplantation events, null findings that remained in subdistribution analysis.With regard to mortality, in subdistribution models, depression symptoms remained a predictor of survival when the competing event of transplantation was considered, an effect which was maintained after *p* value adjustment.

In a subsample where CRP was available, the results remained similar although only continuous depression symptom scores were predictive of mortality. However, these effects become non-significant after applying adjusted *p* value criteria. Controlling for CRP did appear to attenuate the effect of depression as defined by a PHQ-9 cut-off score of ≥10. Depression screening cut-off scores indicate greater levels of depressive affect, which in some cases (but not all) will correspond to clinical depression. CRP and depression have been the focus of past studies, with some showing a positive association between depression and inflammation [22, 23]. This association might explain the observed attenuation of the effect between depression and survival with respect to the PHQ-9 cut-off score, although it should be acknowledged that the association between inflammation and depression in ESKF is mixed [24, 25].

The mechanisms through which depression predicts survival remain elusive, although several candidate pathways exist 26. Depression may impact survival by allostatic dysregulation 26, a potential pathway that implicates the relationship between malnutrition, inflammation and atherosclerosis (MIA syndrome). There are close associations between these factors in ESKF [26–28] and some preliminary evidence that depression could be involved in MIA syndrome 29.

Depression might also be associated with mortality via behavioural mechanisms, most notably treatment non-adherence [26, 30, 31]. Depression is associated with non-adherence to various aspects of the ESKF regimen 31, and non-adherence is predictive of poor outcomes including survival [31, 32]. There is evidence that depression symptoms are associated with increased hospitalisation 5 and health care use 33 in dialysis patients. Although speculative, such increased health care use might be the result of greater non-adherence and poorer self-management in those with depression.

With regard to transplantation, the finding that self-reported depression is not associated with kidney transplantation events is of importance and relevance to clinical practice. Comorbidities are common in dialysis patients and associated with both the eligibility and time to transplantation. Since depression is a prevalent extra renal comorbidity it might be expected to have some association with transplantation events. However, in cause-specific and subdistribution models, there was no evidence of an association between transplant events and depression. It is reassuring that depression symptoms do not differentiate transplant likelihood. However, this is not to say that depression is not important in the context of transplantation, particularly since depressive symptoms predict transplant outcomes (i.e. graft failure, for a review see 34). Our findings partially support past data, which reports no association between depression and transplantation within patients on the transplant waiting list 9. However, depression has been found to be associated with the odds of being on the transplant waiting list 9, with a 5-point increase in depression score associated with a 9% reduction in the odds of being on the waiting list. Unfortunately, wait-listing status data was not available in the current study. However, using competing risks models, we observed no relationship between depression and transplantation across our entire sample. This suggests that the relationship between depression and survival in dialysis patients does not appear to be influenced by fewer transplants in patients with depression and therefore a prolonged time on dialysis, which is associated with poorer outcomes compared with kidney transplantation 10.

Strengths of the study include a large sample size, use of two different validated depression screening tools, very limited data loss (i.e. lost to follow-up) and causal-specific survival models. However, our study has some potential limitations to consider when evaluating the findings. With regard to screening, both the BDI-II and PHQ-9 contain somatic symptoms which overlap with other common symptoms associated with ESKF 35, potentially inflating scores and impacting upon measurement validity. However, both of these tools have been shown to compare well to diagnostic criteria, with validated renal-specific cut-off scores reported in the literature 15. A further limitation was our all-cause mortality end-point, which prevents the evaluation of cause-specific mortality, particularly CVD-related death. Others report an association between cardiovascular-related mortality and depression in dialysis patients 7. A recent study however found that depression was only predictive of all-cause mortality and not CVD-related death 3, albeit when using a higher BDI cut-off score (≥20). Furthermore, due to current complexities of evaluating depression in non-English-speaking patients 36, our study was limited to those who could comprehend the measures; therefore, these findings may not be generalisable to the entire dialysis population. Others have reported moderated effects between depression and ethnicity with respect to both renal disease mortality 21 and all-cause mortality in the general population 37. These findings were not replicated here. However, our reliance on English-speaking patients and our relatively crude *white* vs. *non-white* comparison may have contributed to this null finding; thus, more research examining these effects are warranted.

Lastly, the cut-offs used for the BDII-II and PHQ-9, whilst validated, do not represent a clinical diagnosis of major depressive disorder. Unfortunately, the number of patients who underwent diagnostic interview for MDD as part of the RCT (*n* = 63) was too small to test the association between clinical depression (MDD) and outcomes.

To conclude, our results support past data showing that depression symptoms are associated with mortality in ESKF. Importantly, depressive symptoms do not appear to differentiate between transplantation events. Efforts to further understand the mechanisms through which depression symptoms influence mortality and evaluate which treatment options are the most appropriate for ESKF patients require greater research if outcomes are to be improved.

## Compliance with Ethical Standards

### Conflicts of Interest and Adherence to Ethical Standards

Authors Joseph Chilcot, Ayman Guirguis, Karin Friedli, Michael Almond, Clara Day, Maria Da Silva-Gane, Andrew Davenport, Naomi A Fineberg, Benjamin Spencer, David Wellsted and Ken Farrington declare that they have no conflict of interest. All procedures, including the informed consent process, were conducted in accordance with the ethical standards of the responsible committee on human experimentation (institutional and national) and with the Helsinki Declaration of 1975, as revised in 2000. This article presents independent research funded by the National Institute for Health Research (NIHR) under its Research for Patient Benefit (RfPB) Programme (Grant Reference Number PB-PG-1112-29078). The views expressed are those of the author(s) and not necessarily those of the NHS, the NIHR or the Department of Health.
